# Tumor phenotype and breast density in distinct categories of interval cancer: results of population-based mammography screening in Spain

**DOI:** 10.1186/bcr3595

**Published:** 2014-01-10

**Authors:** Laia Domingo, Dolores Salas, Raquel Zubizarreta, Marisa Baré, Garbiñe Sarriugarte, Teresa Barata, Josefa Ibáñez, Jordi Blanch, Montserrat Puig-Vives, Ana Belén Fernández, Xavier Castells, Maria Sala

**Affiliations:** 1 Department of Epidemiology and Evaluation, IMIM (Hospital del Mar Medical Research Institute), Barcelona, Spain; 2 Research network on health services in chronic diseases (REDISSEC), Barcelona, Spain; 3 General Directorate Public Health, Valencia, Spain; 4 Centre for Public Health Research (CSISP), FISABIO, Valencia, Spain; 5 Galician Breast Cancer Screening Program, Directorate for innovation and management of public health, Santiago de Compostela, Spain; 6 Epidemiology and Assessment Unit UDIAT-Diagnostic Centre, Corporació Sanitària Parc Taulí, Sabadell, Spain; 7 Department of Pediatrics, Obstetrics and Gynecology, Preventive Medicine and Public Health, Universitat Autònoma de Barcelona (UAB), Bellaterra, Spain; 8 Osakidetza Breast Cancer Screening Programme, Basque Country Health Service, Bilbao, Spain; 9 General Directorate of Health Care Programmes, Canary Islands Health Service, Las Palmas de Gran Canaria, Spain; 10 Epidemiology Unit and Girona Cancer Registry, University of Girona, Girona, Spain

## Abstract

**Introduction:**

Interval cancers are tumors arising after a negative screening episode and before the next screening invitation. They can be classified into true interval cancers, false-negatives, minimal-sign cancers, and occult tumors based on mammographic findings in screening and diagnostic mammograms. This study aimed to describe tumor-related characteristics and the association of breast density and tumor phenotype within four interval cancer categories.

**Methods:**

We included 2,245 invasive tumors (1,297 screening-detected and 948 interval cancers) diagnosed from 2000 to 2009 among 645,764 women aged 45 to 69 who underwent biennial screening in Spain. Interval cancers were classified by a semi-informed retrospective review into true interval cancers (n = 455), false-negatives (n = 224), minimal-sign (n = 166), and occult tumors (n = 103). Breast density was evaluated using Boyd’s scale and was conflated into: <25%; 25 to 50%; 50 to 75%; >75%. Tumor-related information was obtained from cancer registries and clinical records. Tumor phenotype was defined as follows: luminal A: ER+/HER2- or PR+/HER2-; luminal B: ER+/HER2+ or PR+/HER2+; HER2: ER-/PR-/HER2+; triple-negative: ER-/PR-/HER2-. The association of tumor phenotype and breast density was assessed using a multinomial logistic regression model. Adjusted odds ratios (OR) and 95% confidence intervals (95% CI) were calculated. All statistical tests were two-sided.

**Results:**

Forty-eight percent of interval cancers were true interval cancers and 23.6% false-negatives. True interval cancers were associated with HER2 and triple-negative phenotypes (OR = 1.91 (95% CI:1.22-2.96), OR = 2.07 (95% CI:1.42-3.01), respectively) and extremely dense breasts (>75%) (OR = 1.67 (95% CI:1.08-2.56)). However, among true interval cancers a higher proportion of triple-negative tumors was observed in predominantly fatty breasts (<25%) than in denser breasts (28.7%, 21.4%, 11.3% and 14.3%, respectively; <0.001). False-negatives and occult tumors had similar phenotypic characteristics to screening-detected cancers, extreme breast density being strongly associated with occult tumors (OR = 6.23 (95% CI:2.65-14.66)). Minimal-sign cancers were biologically close to true interval cancers but showed no association with breast density.

**Conclusions:**

Our findings revealed that both the distribution of tumor phenotype and breast density play specific and independent roles in each category of interval cancer. Further research is needed to understand the biological basis of the overrepresentation of triple-negative phenotype among predominantly fatty breasts in true interval cancers.

## Introduction

The main goal of mammographic screening is to reduce mortality and morbidity from breast cancer through early detection. However, women with interval cancer do not benefit from early detection, as their tumors are detected clinically after a negative screening episode and before the following screening invitation [1].

Interval cancers can be distinguished into four categories by the retrospective review of both screening and diagnostic mammograms: a) true interval cancers are those that showed normal or benign features in the previous screening mammogram; b) false-negative cancers are detected when signs suspicious for malignancy are retrospectively seen on a mammogram; c) minimal-signs are cancers showing detectable but non-specific signs at the latest screening; and d) occult tumors are those that present clinical signs of the disease despite a lack of mammographic abnormalities either at screening or at diagnosis. The European guidelines recommend first reviewing the screening films without histopathological information, and then using the screening and diagnostic films for the definitive classification. This practice involves substantial effort and is not normally routinely performed [1,2]. This explains why there are few large series with specific information on interval cancer categories, especially series providing biological information [3]. Studies evaluating interval cancers and following the recommendations of the European guidelines have found that about half are true interval cancers, over 20% are false negatives [3-5], and fewer than 20% are occult tumors and minimal-sign cancers [5,6].

There is evidence that interval cancers are more likely to have less favorable molecular features than screening-detected cancers, such as a high proportion of tumors not expressing estrogen receptor (ER negative, ER-) or progesterone receptor (PR negative, PR-) [4,7-9]. Some studies have reported a higher proportion of triple-negative cancers (ER-, PR-, human epidermal growth factor receptor 2 (HER2)-) among interval cancers [7,10] and this increase is even higher if only the subset of true interval cancers is considered in comparison to screening-detected cancers [4]. So far, this tumor phenotype lacks the benefit of specific adjuvant therapy and is associated with an aggressive behavior pattern and poor prognosis [11].

Breast density has also been related to interval cancer. There is increasing evidence that women with dense breasts are more likely to be diagnosed with interval cancer [12-14], but the role of breast density has not yet been elucidated [13,15]. A masking effect, which would contribute to hide the tumors [15], as well as a biological effect related to tumor growth [16], has been purposed. Because breast density influences both the risk and detection of breast cancer, as well as the likelihood of developing certain pathological subtypes [17,18], studying this factor in interval cancers would be of great interest.

We hypothesized that the roles of tumor phenotype and that of breast density differ in distinct categories of interval cancers. The aim of this study was to describe the tumor-related characteristics of true interval cancers, false negatives, minimal-sign cancers and occult tumors, and to assess the association of breast density and tumor phenotype in the four interval cancer categories. This study provides a comprehensive approach to the four categories of interval breast cancer identified from one of the largest cohorts of women participating in population-based breast cancer screening.

## Methods

### Setting

We performed a case–control study nested in a cohort of 645,764 women aged 45 to 69 years, screened in Spain between 1 January 2000 and 31 December 2006, and followed up until June 2009. These women underwent a total of 1,508,584 screening mammograms. During the study period, 5,309 cancers were detected in routine screening mammograms and 1,669 emerged as interval cancers, including both invasive and *in situ* carcinomas.

All women resident in Spain aged 50 to 69 years are actively invited to participate in the population-based screening program by a written letter every 2 years, following the European guidelines for Quality Assurance in Mammographic Screening Recommendations [1]. This nationwide program achieves the required standards [19].

We gathered data from five Spanish regions (Basque Country, Canary Islands, Catalonia, Galicia, and Valencia), covering a population of 752,487 women in 2005. Two mammographic projections (mediolateral-oblique and craniocaudal views) were made both in the initial and in successive rounds, except in one program. All mammograms were read by two radiologists, except in two programs, and the classification used for mammogram reading was BI-RADS [20]. Two regions switched to digital mammography during 2003 to 2005.

All screening programs keep mammography registers with data from participants and the final outcome of screening. Once a tumor is histologically confirmed, the woman is referred to a hospital for treatment and follow up. They are not further invited to screening, as they are controlled in the health care system.

Study data were collected using a protocol approved by the ethics committee of Parc de Salut Mar (CEIC-Parc de Salut MAR), Barcelona. Specific patient consent was not required because we used retrospective data from screening participants who had previously signed information release documents.

### Study population: case and control definitions

Case subjects with interval cancer and control subjects with screening-detected cancer were drawn from women enrolled in any of the screening programs. We used the definition of interval cancers purposed in the European guidelines: “primary breast cancer arising after a negative screening episode, with or without further assessment, and before the next invitation to screening, or within 24 months for women who reached the upper age limit” [1]. The overall 1,669 interval cancers were matched by screening program and the year of the last screening mammogram to one screening-detected cancer, that is, a pathologically-confirmed malignant lesion identified during the screening process. We excluded those cases and controls with no available information on screening and diagnostic (only for interval cancers) mammograms. Finally, we analyzed 948 interval cancers, and 1,297 screening-detected cancers. Ductal *in situ* carcinomas were excluded from the analysis.

### Assessment of interval cancers and breast density classification

Interval cancers were identified by merging data from the registers of screening programs with population-based cancer registries, the regional Minimum Basic Data Set (MBDS) and hospital-based cancer registries. The use of different data sources ensured the quality and homogeneity of the process across the study period and regions. Population-based cancer registries covered four out of five regions. The MBDS (based on hospital discharges with information on the principal diagnosis) is updated yearly and is available in all regions. All data sources kept information on the time of diagnosis, which allowed us to ensure that all interval cancers fitted the case definition.

For interval cancer classification, three panels with three experienced radiologists performed a semi-informed retrospective review of both screening and diagnostic mammograms through independent double reading with arbitration. Screening mammograms were first reviewed alone, without the radiologists seeing the diagnostic mammogram and without histological information (blind review). Interval cancers were provisionally classified into positive (abnormality clearly visible and warrants assessment), negative (normal mammogram), and minimal-sign (subtle abnormality, not necessarily regarded as warranting assessment). Later, the diagnostic and screening mammograms were reviewed together and interval cancers were definitively classified into true interval cancers, false negatives, minimal-sign cancers, and occult tumors [1]. In the definitive classification, we ensured that the site where the minimal signs were identified correlated with the site of the interval cancer. When there was no correlation, the case was considered a true interval cancer.

One radiologist from each panel determined the breast density of the cancer-free breast, for both interval and screening-detected cancers. Breast density was evaluated using Boyd’s scale, a semiquantitative score of six categories using percentages of density: A: 0%; B: 1 to 10%; C: 10 to 25%; D: 25 to 50%; E: 50 to 75%; F: 75 to 100% [21]. For purposes of assessing the impact of predominately fatty versus increasingly dense breasts, the first three categories were combined into the <25% group [22].

### Study variables

The woman’s age at diagnosis was obtained from the date of birth and date of the screening mammogram. Tumor-related information (the tumor histology, grade, size, lymph node involvement, and ER, PR, HER2, p53 and Ki67 status) was obtained from the cancer registries, hospital-based registers, and from the clinical records. Biomarker assessment was performed as part of the diagnostic process in the hospitals. The positivity criteria used by each hospital followed international recommendations and their updates throughout the study period [23,24]. Tumors were considered positive when more than 20% and 10% of cells stained positive for Ki67 and p53, respectively. For the histological classification, we used ICD-O, 3rd edition. Histological grade was defined according to the Scarff-Bloom-Richardson criteria, modified by Elson [25].

Based on the expression of ER, PR and HER2, tumors were classified into four phenotypes: 1) luminal A: ER+/HER2- or PR+/HER2-; 2) luminal B: ER+/HER2+ or PR+/HER2+; 3) HER2: ER-/PR-/HER2+; and 4) triple-negative: ER-, PR-, HER2- [26].

### Statistical analysis

Comparisons were established between screening-detected cancers, true interval cancers, false negatives, minimal-sign cancers, and occult tumors. Statistical significance was assessed using the Chi-square or Fisher exact test for categorical variables, and one-way analysis of variance (ANOVA) for continuous variables. If a significant difference was found, we calculated standardized Pearson residuals as a measure of deviation between the observed and expected values to determine which cells contributed most to the Chi-square estimator [27]. Clinical features, age at diagnosis, breast density, biomarker expression, and the phenotypic classification were compared between study groups. Then, we carried out a stratified analysis of tumor phenotype and breast density by study groups.

A multinomial regression analysis was computed to determine the effect of tumor phenotype and breast density on the odds of developing a true interval cancer, a false negative, a minimal-sign cancer, or an occult tumor versus screening-detected cancers. Our final multinomial regression model was adjusted for screening program (categorical), age (continuous), and tumor size (categorical, <11 mm; 11 to 20 mm; 21 to −50 mm; >50 mm). The outputs were plotted, showing the adjusted odds ratio (OR) and the 95% CI for each category of interval cancer, which served as the endpoints of the multinomial model.

We conducted sensitivity analyses by including or excluding screening-detected cancers diagnosed in prevalent screening. We tested different reference categories for breast density (≤10%, ≤50%), and we checked the inclusion of covariates into the multivariate models (year of screening mammogram, histological grade, Ki67 and p53 status, the use of digital or analog mammography, and menopausal status). The sensitivity analyses showed no significant differences with respect to the definitive multinomial model. We examined the interaction between breast density and phenotype and found a non-significant effect within the multiple endpoints of the multinomial model.

All *P*-values were based on two-sided tests and were considered statistically significant if <0.05. Statistical analyses were performed using the SPSS (version 12.0) and R statistical software programs.

## Results

A total of 1,297 screening-detected cancers and 948 interval cancers were included in the analyses. Most interval cancers were true interval cancers (n = 455, 48.0%), followed by false negatives (n = 224, 23.6%), minimal-sign cancers (n = 166, 17.5%) and occult tumors (n = 103, 10.9%).

Table 1 summarizes information on age at diagnosis and tumor-related characteristics of screening-detected cancers and interval cancer categories. Women with true interval cancers and occult tumors were younger (mean age 56.4 years and 55.1 years, respectively) than women in the remaining subsets (*P* <0.001). Over 80% of true interval cancers were detected 12 months after the last screening or later, whereas 42.7% of occult tumors developed within the first 12 months. As expected, the highest percentage of tumors ≤10 mm in size was found among screening-detected cancers (34.8%; *P* <0.001). Among interval cancers, the percentage ranged from 7.9 to 13.3% in true interval cancers and occult tumors, respectively. Extremely dense breasts (>75%) were most frequently associated with occult tumors followed by false-negative cancers and true interval cancers (28.2, 17.0 and 16.5%, respectively, versus 11.6% in screening-detected cancers; *P* <0.001).

**Table 1:** **Comparison of age at diagnosis and tumor characteristics at diagnosis between screening-detected cancers (n = 1,297) and interval cancers (n** = **948)**

	**Screening-detected cancers**	**True interval cancers**	**False negatives**	**Minimal-sign cancers**	**Occult tumors**	
	**n = 1,297**	**n = 455**	**n = 224**	**n = 166**	**n = 103**	***P***-**value**^**†**^
**Interval cancer entities, n (%)**^‡^		455 (48.0)	224 (23.6)	166 (17.5)	103 (10.9)	
**Time since last screening, n (%)**						
<=12 months		89 (19.6)	73 (32.7)	53 (32.1)	44 (42.7)	
>12 months		364 (80.4)	150 (67.3)	112 (67.9)	59 (57.3)	<0.001
**Age, y, mean (95% CI)**	57.6 (57.3, 57.9)	56.4 (55.9, 57.0)	57.4 (56.6, 58.1)	56.8 (56.0, 57.6)	55.1 (54.0, 56.2)	<0.001
**Tumor size, mm, mean (95% CI)**	15.7 (15.1, 16.3)	25.3 (23.6, 26.9)	23.9 (22.1, 25.8)	22.7 (20.5, 24.8)	19.3 (17.0, 21.6)	<0.001
**Focality, n (%)**						
Unifocal	1030 (82.8)	341 (79.1)	171 (78.4)	118 (74.7)	83 (85.6)	
Multifocal and/or multicentric	214 (17.2)	90 (20.9)	47 (21.6)	40 (25.3)	14 (14.4)	0.041
Unknown	53	24	6	8	6	
**Tumor size, n (%)**						
<= 10 mm	452 (34.8)*	36 (7.9)*	18 (8.0)*	22 (13.3)*	13 (12.6)*	
11 to 20 mm	521 (40.2)	147 (32.3)	79 (35.3)	53 (31.9)	45 (43.7)	
21 to 50 mm	233 (18.0)*	171 (37.6)*	78 (34.8)*	62 (37.3)*	23 (22.3)	
>50 mm	91 (7.0)*	101 (22.2)*	49 (21.9)*	29 (17.5)	22 (21.4)*	<0.001
Unknown	0	0	0	0	0	
**Lymph node involvement, n (%)**						
Negative	872 (70.2)*	195 (50.4)*	102 (54.5)	76 (49.7)*	54 (62.1)	
Positive	371 (29.8)*	192 (49.6)*	85 (45.5)	77 (50.3)*	33 (37.9)	<0.001
Unknown	54	68	37	13	16	
**Histological type, n (%)**						
Ductal	1039 (80.5)	349 (77.6)	165 (74.0)	129 (77.7)	70 (68.6)	
Lobular	109 (8.4)*	54 (12.0)	36 (16.1)*	16 (9.6)	21 (20.6)*	
Other	143 (11.1)	47 (10.4)	22 (9.9)	21 (12.7)	11 (10.8)	<0.001
Unknown	6	5	1	0	1	
**Histological grade, n (%)**						
I	390 (34.9)*	57 (14.9)*	41 (21.4)	33 (22.9)	19 (22.6)	
II	474 (42.4)	149 (39.0)	88 (45.8)	62 (43.1)	42 (50.0)	
III	241 (21.6)*	171 (44.8)*	61 (31.8)	47 (32.6)	20 (23.8)	
NA	13 (1.2)	5 (1.3)	2 (1.0)	2 (1.4)	3 (3.6)	<0.001
**Breast density, n (%)**						
<25%	510 (39.3)*	139 (30.5)*	81 (36.2)	64 (38.6)	15 (14.6)*	
25 to 50%	359 (27.7)	127 (27.9)	60 (26.8)	47 (28.3)	20 (19.4)	
51 to 75%	277 (21.4)*	114 (25.1)	45 (20.1)	39 (23.5)	39 (37.9)*	
>75%	151 (11.6)	75 (16.5)	38 (17.0)	16 (9.6)	29 (28.2)*	<0.001
Unknown	0	0	0	0	0	

Missing values were excluded from the calculations of percentages. *Standardized Pearson residuals with statistically significant deviation between observed and expected values.

^†^*P*-values for comparison of characteristics among the five study groups were obtained by one-way analysis of variance for continuous variables and the Chi-square test for categorical variables. All tests were two-sided. ^‡^Row percentages.

The expression of biomarkers among study groups is detailed in Table 2. True interval cancers were less likely to express ER and PR than screening-detected cancers but were more likely to overexpress HER2, p53, and Ki67. In contrast, the molecular profile observed among occult tumors revealed a higher percentage of ER + cancers (88.4 versus 82.5%) and a lower percentage of HER2+ cancers (14.1 versus 21.9%) compared with screening-detected cancers. Molecularly, false-negative tumors were similar to screening-detected cancers, although they showed a higher proportion of tumors overexpressing Ki67 (50.3 versus 40.2%). Almost 35% of minimal-sign cancers overexpressed p53.

**Table 2:** Biomarker expression among screening-detected cancers (n = 1,297) and distinct categories of interval cancers (n = 948)

	**Screening-detected cancers**	**True interval cancers**	**False negatives**	**Minimal-sign cancers**	**Occult tumors**	
	**n = 1,297**	**n = 455**	**n = 224**	**n = 166**	**n = 103**	***P*-value**^**†**^
**Estrogen receptor**	1022 (82.5)*	283 (63.2 )*	178 (81.7)	114 (71.3)	84 (88.4)	<0.001
Missing values	58	7	6	6	8	
**Progesterone receptor**	775 (63.7)*	214 (48.2)*	128 (59.0)	86 (54.4)	60 (64.5)	<0.001
Missing values	81	11	7	8	10	
**HER2**	203 (21.9)	113 (29.1)*	44 (24.0)	31 (23.1)	11 (14.1)	0.018
Missing values	371	67	41	32	25	
**p53**	149 (22.7)	86 (36.6)*	23 (21.5)	27 (34.6)	17 (28.8)	<0.001
Missing values	641	228	117	88	44	
**Ki67**	381 (40.2)	169 (52.5 )*	83 (50.3)	50 (41.7)	26 (39.4)	0.001
Missing values	349	133	59	46	37	

Number of cases and percentage of tumors with positive biomarker expression. *Standardized Pearson residuals with statistically significant deviation between observed and expected values. ^†^*P*-values for comparison of characteristics among the five study groups were obtained by two-sided Chi-square test. HER2, human epidermal growth factor receptor 2.

The distribution of tumor phenotypes among study groups is shown in Table 3. True interval cancers and minimal-sign tumors showed a higher proportion of triple-negative cancers (19.9 and 17.3%, respectively), whereas false-negative and occult tumors showed a similar tumor phenotype profile to screening-detected cancers.

**Table 3:** Distribution of tumor phenotypes among screening-detected cancers (n = 1,297) and categories of interval cancers (n = 948)

	**Screening-detected cancers**	**True interval cancers**	**False negatives**	**Minimal-sign cancers**	**Occult tumors**	
	**n = 1,297**	**n = 455**	**n = 224**	**n = 166**	**n = 103 (%)**	**P value**^**†**^
**Tumor phenotype**						
Luminal A	629 (68.3)	197 (50.9)*	124 (68.1)	79 (59.4)	62 (79.5)	
Luminal B	139 (15.1)	60 (15.5)	29 (15.9)	18 (13.5)	8 (10.3)	
HER2	62 (6.7)	53 (13.7)*	14 (7.7)	13 (9.8)	3 (3.8)	
Triple-negative	91 (9.9)*	77 (19.9)*	15 (8.2)	23 (17.3)	5 (6.4)	<0.001
Unknown	376	68	42	33	25	

Results are expressed as number (%). Tumor phenotype = Luminal A (ER+/HER2- or PR+/HER2-); Luminal B (ER+/HER2+ or PR+/HER2+); HER2 (ER-/PR-/HER2+); Triple-negative (ER-/PR-/HER2-). *Standardized Pearson residuals with statistically significant deviation between observed and expected values. ^†^The distribution of tumor phenotype was compared among the study groups using the two-sided Chi-square test. ER, estrogen receptor; PR, progesterone receptor; HER2, human epidermal growth factor receptor 2.

In Table 4 is shown the distribution of tumor phenotypes among study groups, stratified by breast density. According to breast density, differences in phenotype distribution were statistically significant among true interval cancers. The highest proportion of triple-negative cancers among true interval cancers was observed in breasts with 25% lower density than in denser breasts (28.7, 21.4, 11.3 and 14.3%, respectively; *P* <0.001).

**Table 4:** Distribution of tumor phenotypes among screening-detected cancers (n = 1,297) and categories of interval cancers (n = 948) stratified by breast density

	**Breast density**				
	**<25%**	**25 to 50%**	**50 to 75%**	**>75%**	***P*-value**^**†**^
**Tumor phenotype**					
**Screening-detected cancers**					
Luminal A	247 (68.6)	165 (65.5)	142 (70.3)	75 (71.4)	
Luminal B	51 (36.7)	49 (19.3)	24 (11.9)	15 (14.3)	
HER2	20 (5.6)	16 (6.3)	17 (8.4)	9 (8.6)	
Triple negative	42 (11.7)	24 (9.4)	19 (9.4)	6 (5.7)	
Unknown	150	105	25	46	0.306
**True interval cancers**					
Luminal A	60 (52.2)	51 (45.5)	49 (50.5)	37 (58.7)	
Luminal B	13 (11.3)	17 (15.2)	15 (15.5)	15 (23.8)	
HER2	9 (7.8)	20 (17.9)	22 (22.7)*	2 (3.2)*	
Triple negative	33 (28.7)*	24 (21.4)	11 (11.3)	9 (14.3)	
Unknown	24	15	17	12	<0.001
**False negatives**					
Luminal A	40 (62.5)	39 (75.0)	22 (64.7)	23 (71.9)	
Luminal B	10 (15.6)	4 (7.7)	9 (26.5)	6 (18.8)	
HER2	6 (9.4)	4 (7.7)	2 (5.9)	2 (6.3)	
Triple negative	8 (12.5)	5 (9.6)	1 (2.9)	1 (3.1)	
Unknown	17	8	11	6	0.375
**Minimal-sign cancers**					
Luminal A	30 (60.0)	26 (66.7)	16 (55.2)	7 (46.7)	
Luminal B	6 (12.0)	3 (7.7)	5 (17.2)	4 (26.7)	
HER2	2 (4.0)	5 (12.8)	2 (6.9)	4 (26.7)	
Triple negative	12 (24.0)	5 (12.8)	6 (20.7)	0 (0)	
Unknown	14	8	10	1	0.081
**Occult tumors**					
Luminal A	8 (80.0)	12 (80.0)	26 (78.8)	16 (80.0)	
Luminal B	0 (0)	2 (13.3)	4 (12.1)	2 (10.0)	
HER2	0 (0)	1 (6.7)	0 (0)	2 (10.0)	
Triple negative	2 (20.0)	0 (0)	3 (9.1)	0 (0)	
Unknown	5	5	6	9	0.296

Results are expressed as number (%). Tumor phenotypes = Luminal A (ER+/HER2- or PR+/HER2-); Luminal B (ER+/HER2+ or PR+/HER2+); HER2 (ER-/PR-/HER2+); Triple-negative (ER-/PR-/HER2-). *Standardized Pearson residuals with statistically significant deviation between observed and expected values. ^†^*P*-value assesses the distribution of tumor phenotype distribution among breast density categories within study groups, using the two-sided Chi-square test, or Fisher exact test when appropriate. ER, estrogen receptor; PR, progesterone receptor; HER2, human epidermal growth factor receptor 2.

**Figure 1:**
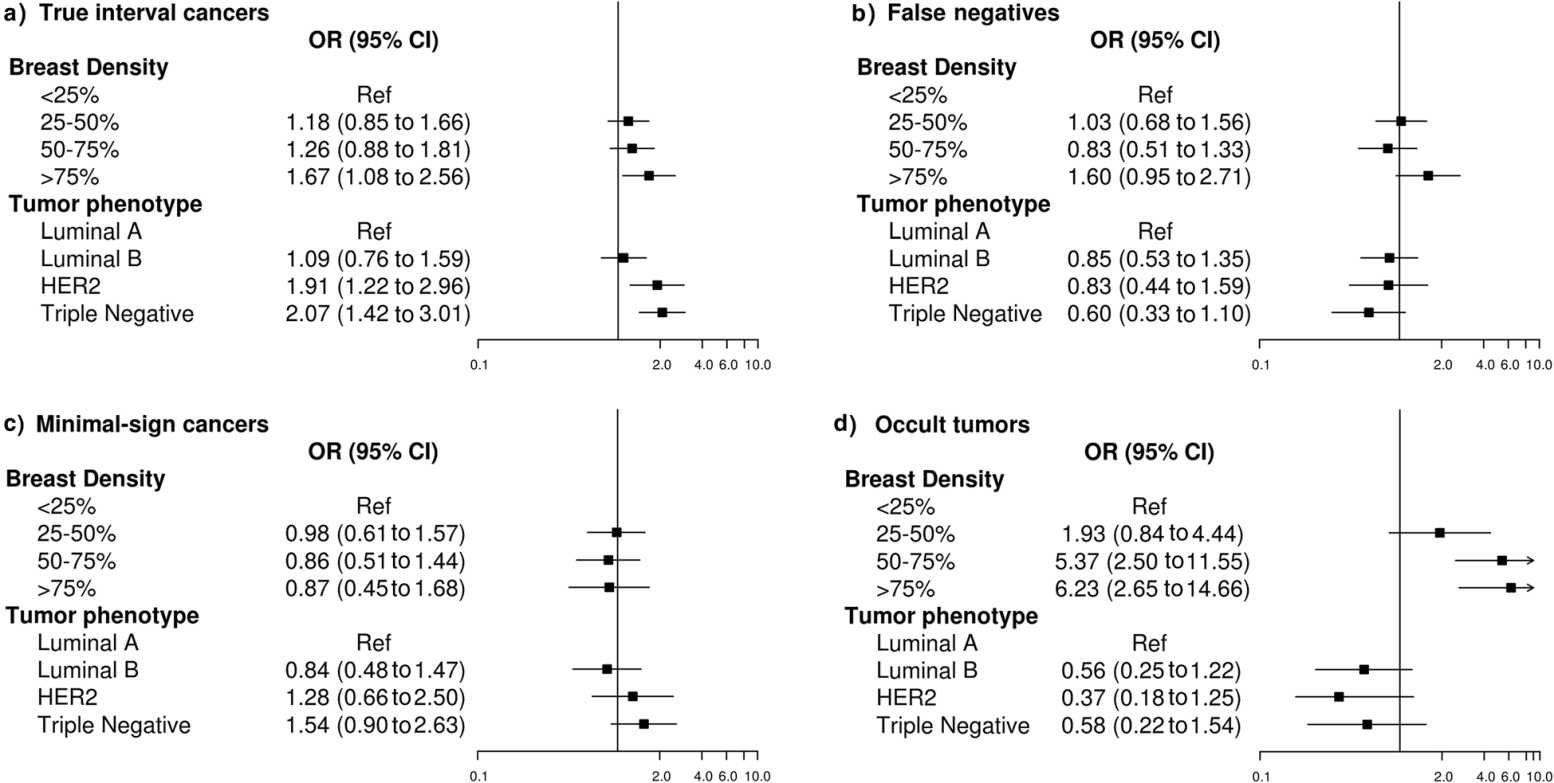
**Multinomial logistic regression model of the association of breast density and tumor phenotypes with categories of interval cancer, adjusted for age at screening, screening program, and tumor size.** The association of breast density with tumor phenotype, adjusted by screening program (categorical), age (continuous), and tumor size (categorical, <11 mm; 11 to 20 mm; 21 to 50 mm; >50 mm), is shown for the multiple endpoints of the multinomial logistic regression models, which are **(a)** true interval cancers; **(b)** false negatives; **(c)** minimal-sign cancers and **(d)** occult tumors. The reference category (Ref) is screening-detected cancers. The black squares and the horizontal lines represent the odds ratios (OR) and corresponding 95% CI, respectively. ORs are presented on the log scale. Tumor phenotype = Luminal A: ER/HER2- or PR+/HER2-; Luminal B: ER+/HER2+ or PR+/HER2+; HER2: ER-/PR-/HER2+; Triple-negative: ER-/PR-/HER2-.

Adjusted OR and 95% CI estimated by multinomial regression analysis are plotted in Figure 1. True interval cancers were associated with HER2 and triple-negative phenotypes (OR 1.91, 95% CI 1.22, -2.96; OR 2.07, 95% CI 1.42, 3.01, respectively) and extremely dense breasts (OR 1.67, 95% CI 1.08, 2.56). Occult tumors were over six times more likely to develop in extremely dense breasts (OR 6.23, 95% CI 2.65, 14.66). False-negative cancers showed a non-significant tendency to occur in extremely dense breasts, whereas in the adjusted model, minimal-sign cancers showed no association with either breast density or tumor phenotype.

## Discussion

This comprehensive study suggests that true interval and minimal-sign cancers showed similar tumor phenotype distribution, with almost 20% of these tumors being triple negative. In contrast, false-negative and occult tumors were phenotypically closer to screening-detected cancers. High breast density was mainly associated with occult tumors, and to a lesser extent, to true interval cancers and false negatives. However, among true interval cancers, those with the triple-negative phenotype were more likely to occur in predominately fatty breasts than in extremely dense breasts.

The proportion of tumors classified as true interval cancers, false negatives, minimal-sign cancers, or occult tumors is in line with previous series [3-6], that followed the European guidelines for the definition and classification of interval cancers. The percentage of false negatives slightly exceeded the limit of 20% recommended by the European guidelines, but is lower than that in other contexts [3,28]. Nevertheless, the lack of a standardized method for the radiological classification of interval cancers, together with the subjective nature of mammography interpretation, hamper valid comparisons between screening programs [2,28].

As expected by the lead time, all interval cancers were larger at diagnosis and were more likely to show lymph node involvement than screening-detected cancers. In agreement with previous work [4,8], true interval cancers were those with the longest waiting time to breast cancer diagnosis and were also the largest. However, some studies that analyzed occult tumors and true interval cancers together have reported that the clinical features of this subset differed less than those of screening-detected tumors [8]. As occult tumors were those detected earliest after screening, resulting in a higher proportion of small carcinomas and showing a molecular pattern similar to screening-detected cancers, grouping true interval and occult tumors together may lead to underestimation of the less prognostically favorable features of true interval cancers.

True interval and minimal-sign cancers showed similarities in their patterns of biomarker expression and tumor phenotype. Our results confirm that true interval cancers were less likely to express hormonal receptors [4,8,9,29] and support previous series reporting overexpression of HER2, p53, and Ki67 [4,9,30]. To our knowledge, this is the first study that provides complete molecular characterization of minimal-sign cancers. In line with previous evidence for the overrepresentation of triple-negative tumors among interval cancers [4,7,10], we found that most triple-negative tumors were concentrated among true interval and minimal-sign cancers. The biological similarities shared by both entities suggest that some minimal-sign tumors could be a more advanced form of true interval cancer, whereas false negatives seem to be a clearly distinct entity from minimal-sign cancers. These two entities should not be classified together, as has been done in some previous studies [31,32].

Breast density is a well-known risk factor for breast cancer and particularly interval cancer [13,14], but its association with tumor phenotypes remains controversial. Our findings revealed that luminal cancers were more likely to be detected in extremely dense breasts than in predominately fatty breasts, in agreement in with some previous studies [10,33,34], but contrasting with others [18]. Yanhjyan *et al*. [18] reported a higher proportion of triple-negative cancers among women with dense breasts. However, their study design was not comparable with ours, as these authors did not take into account whether the cancers were detected by screening. Unless the detection mode is considered, the association of triple-negative cancers and breast density may be overestimated, because tumors detected between two screenings are more likely to be detected in women with dense breasts and to be triple negative [15,17].

Our findings support the association of breast density and interval cancer independently of phenotype. The association of breast density and true interval cancers reinforces the hypothesis that some tumors are stimulated by growth factors found in dense breasts [35]. However, the overrepresentation of triple-negative tumors among predominantly fatty breasts in true interval cancers may reflect the aggressive behavior, rapid carcinogenesis and nonlinear progression of this tumor phenotype, regardless of breast density [11,36]. Further research is still needed to understand the biological basis of the association of breast density and tumor phenotypes, taking into account the mode of detection. The knowledge of epidemiological factors and radiological features predictive of an aggressive tumor subtype, such as the triple-negative phenotype, could add information for future personalized screening programs in women at risk of interval cancer.

The strong association of breast density and occult tumors pointed to a masking effect, confirming the assumptions noted years ago by Houssami [2]. Our findings also reinforce the idea that a masking effect mainly affects cancers that developed up to 12 months after screening [15]. Nevertheless, breast density appears to play a lesser role in false negatives, in line with previous series [13,37]. Breast density remains a major issue in breast cancer screening because it is one of the variables proposed to tailor screening [38]. Information on its role among interval cancer categories along with data on its relationship with tumor phenotypes may be useful to estimate the potential benefit of personalizing screening strategies on the basis of this factor.

The strengths of the current study are the large sample size and the completeness of the information. These factors have allowed us to study the role of breast density and tumor phenotype for each interval cancer category and to describe some features that may help to better understand their etiology.

There are, however, some limitations that should be considered. First, misclassification among interval cancers cannot be excluded. Some interval cancers could be classified as screening-detected if symptomatic women waited for the screening visit instead of making an immediate appointment with a physician. However, such misclassification would attenuate differences in tumor characteristics among study groups. Second, not all cases would have been phenotypically classified. Since this lack of information affects both screening-detected cancers and interval cancers, and was similar in all screening programs we do not believe that it affects the results. However, data on p53 and Ki67 were not always available because they were not routinely checked in all centers. Given that their lack of availability was not random, these data were not entered into the multinomial model. Third, grouping breast density into four categories reduced the sample size in the stratified analyses, but allowed the role of extremely dense breasts to be assessed. Collapsing breast density into two categories (≤50 and >50%) diminished the magnitude of the association of breast density and distinct categories of interval cancer (data not shown). Fourth, some important variables associated with breast density, such as body mass index, age at menarche or childbirth, are not routinely collected by screening programs, and therefore we could not adjust for these potential confounders.

## Conclusions

Our findings revealed that both the distribution of tumor phenotype and breast density play specific and independent roles in each category of interval cancer. Almost half of the interval cancers were true interval cancers, which encompassed a high percentage of tumors with a molecular profile associated with poor prognosis on the one hand and were more likely to be detected among women with extremely dense breasts on the other. False-negative and occult tumors had similar phenotypic characteristics to screening-detected cancers, high breast density being strongly associated with occult tumors. Minimal-sign cancers were biologically close to true interval cancers but showed no association with breast density. In view of the heterogeneity within interval cancers, further studies aiming to characterize interval cancers should avoid grouping true interval cancers and occult tumors, or false-negative and minimal-sign cancers. Knowledge of the clinical and biological particularities of interval cancers and of the role of breast density may be useful for the design of new risk-based screening strategies.

## Abbreviations

ANOVA: analysis of variance; CI: confidence intervals; ER: estrogen receptor; HER2: human epidermal growth factor receptor 2; MBDS: Minimum Basic Data Set OR: odds ratio; PR: progesterone receptor.

## Competing interests

The authors declare that they have no competing interests.

## Authors’ contributions

LD drafted the first version of the manuscript, performed the statistical analysis, and participated in the design of the study. MS, DS, RZ, and XC, conceived the study and participated in its design and coordination, and critically revised the manuscript for important intellectual content. MB, GS, TB, JI, JB, LD, MPV and ABF participated in the acquisition of data from screening programs and from clinical records, helped in the interpretation of the results, and helped to draft the manuscript. JB supported the statistical analysis, gathered data, and validated the whole database. ABF, JI, LD, DS and MS coordinated the review process for interval cancer classification and for the assessment of breast density. All authors critically reviewed the manuscript. All of them read and approved the final manuscript.
